# NiFe_2_O_4_/ZnO-coated Poly(L-Lactide) nanofibrous scaffold enhances osteogenic differentiation of human mesenchymal stem cells

**DOI:** 10.3389/fbioe.2022.1005028

**Published:** 2022-10-17

**Authors:** Shiva Shariati, Ehsan Seyedjafari, Fatemeh Sadat Mahdavi, Amirhosein Maali, Elaheh Ferdosi-Shahandashti

**Affiliations:** ^1^ Department of Medical Biotechnology, School of Medicine, Babol University of Medical Sciences, Babol, Iran; ^2^ Department of Medical Biotechnology, School of Advanced Medical Sciences, Golestan University of Medical Sciences, Golestan, Iran; ^3^ Student Research Committee, Babol University of Medical Sciences, Babol, Iran; ^4^ Department of Biotechnology, College of Sciences, University of Tehran, Tehran, Iran; ^5^ Department of Immunology, Pasteur Institute of Iran, Tehran, Iran; ^6^ Department of Medical Biotechnology, Faculty of Allied Medicine, Qazvin University of Medical Sciences, Qazvin, Iran; ^7^ Cellular and Molecular Biology Research Center, Health Research Institute, Babol University of Medical Sciences, Babol, Iran

**Keywords:** nanocomposites, adipose tissue-derived mesenchymal stem cell, poly-l-lactide, osteogenesis, cell differentiation

## Abstract

**Background:** A combination of bioceramics and polymeric materials has attracted the research community’s interest in bone tissue engineering. These composites are essential to support cell attachment, proliferation, and osteogenesis differentiation, which are vital as a classic strategy in bone tissue engineering. In this study, NiFe2O4/ZnO-coated poly L-Lactide (PLLA) was employed as a scaffold to evaluate the osteogenic differentiation capability of human adipose tissue derived mesenchymal stem cells (hAMSCs).

**Material and methods:** The electrospun PLLA nanofibers were fabricated, coated with nanocomposite (NiFe2O4/ZnO), and evaluated by the water contact angle (WCA), tensile test, attenuated total reflectance fourier-transform infrared (ATR-FTIR) and scanning electron microscopy (SEM). Then, the osteogenic differentiation potential of hAMSCs was assessed using NiFe2O4/ZnO-coated PLLA compared to tissue culture plastic (TCP) and a simple scaffold (PLLA) *in vitro* conditions.

**Results:** The adhesion, proliferation, and differentiation of hAMSCs were supported by the mechanical and biological properties of the NiFe2O4/ZnO-coated PLLA scaffold, according to SEM and 4′,6-Diamidino-2-phenylindole dihydrochloride (DAPI) staining patterns. During bone differentiation, Alkaline phosphatase (ALP) enzyme activity, biomineralization, calcium content, and osteogenic gene expression (ALP, Osteonectin, Osteocalcin, Collagen type I, and Runx2) were higher on NiFe2O4/ZnO-coated PLLA scaffold than on PLLA scaffold and TCP.

**Conclusion:** Based on our results, the osteogenic differentiation of hAMSCs on the improved biological scaffold (PLLA coated with NiFe2O4/ZnO) could accelerate due to the stimulating effect of this nanocomposite.

## Graphical Abstract

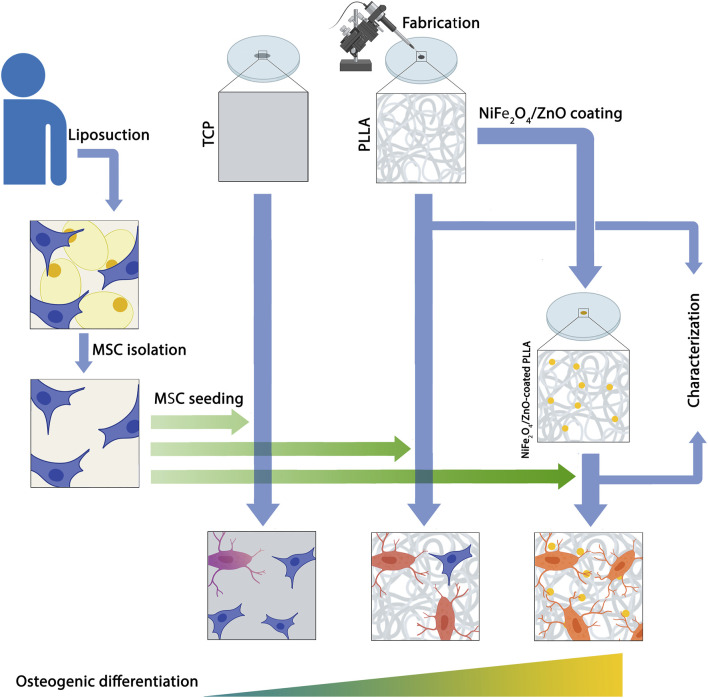

## 1 Highlights

• Bone tissue engineering is a dynamic, regenerative medicine procedure that aims to provide structural support for cell development, proliferation, and adhesion, as well as growth factors or other active substances.
• One scaffold frequently employed for this purpose is Poly (L-lactide) or (PLLA), an aliphatic thermoplastic polyester.
• It is assumed that magnetic nanostructures could bind to cell surfaces, regulating cell function and increasing bone cells activity, resulting in bone tissue regeneration.
• In this study, a NiFe_2_O_4_/ZnO-coated PLLA scaffold was used to assess the osteogenic differentiation capability of human adipose tissue-derived Mesenchymal Stem Cells (hAMSCs) with the aim of bone tissue regeneration *in vivo* condition.
• The differentiation of hAMSC into the osteogenic lineage demonstrates that NiFe_2_O_4_/ZnO coated PLLA could provide a proper support role by mimicking the ECM architecture and culminating in osteoblast adhesion, proliferation, differentiation, and maturation.
• It is thought that NiFe_2_O_4_/ZnO-coated PLLA could have favorable magnetic and mechanical properties and serves as a proper platform for directing connections and cellular activity toward the osteogenic lineage

## 2 Introduction

Natural bone is a dynamic and multifaceted organ with the hierarchical and architectural arrangement of nanoscale to microscopic dimensions that performs fundamental biological functions such as body mobility, organ protection, and regulation of homeostasis of the hematopoietic cell, etc., (Ye et al., 2020; Zhang et al., 2020). Repairing and regenerating bone tissue depends on the injury’s extent, which can adequately repair slight injuries, such as microcracks and some minor fractures. Nevertheless, bone fractures that exceed the threshold (more than 2 cm) are beyond the capacity of this tissue, which can be induced by trauma, congenital disabilities, tumor resection, and other causes (Koons et al., 2020). Depending on the anatomical location, it cannot be entirely and permanently repaired without clinical intervention (Yousefi et al., 2016; Koons et al., 2020).

Bone tissue engineering (BTE) is a dynamic and regenerative medicine process that establishes structural support for cell growth, proliferation, and adhesion through growth factors or active substances. BTE is critical for accelerating differentiation and extracellular matrix (ECM) development. It can potentially integrate and regenerate a specific functional tissue compared to conventional approaches (Baino et al., 2015; Kumar et al., 2018; Porgham Daryasari et al., 2019). Scaffolds provide surfaces that promote stem cell cohesion, survival, migration, proliferation, and differentiation. Also, their porous structure facilitates the formation of arteries, angiogenesis, and a bone-like environment, which results in bone tissue regeneration by mimicking the configuration of ECM (Yousefi et al., 2016; Kumar et al., 2018).

PLLA, an aliphatic thermoplastic polyester, is a frequently employed scaffold for mimicking the bone ECM structure (Kumar et al., 2018). This hydrophilic polymer with electrospinning fabrication is a cost-effective method that is widely used in tissue engineering (Tavangar et al., 2018; Porgham Daryasari et al., 2019). PLLA nanofibers with a high surface-to-volume ratio and a high porosity mimic the role of the bone ECM, promoting hydroxyapatite production, mineral deposition, and optimum vascular integration. Additionally, its strength and stiffness provide a proper framework for developing osteogenic progenitor cells and bone conduction (Liu et al., 2016; Tavangar et al., 2018). Despite their benefits in tissue engineering, Pure PLLA stereoisomers have low biologic activity and surface characteristics. Mixing nanoparticles with a polymer matrix to replicate the architecture of bone nanocomposites makes it possible to increase their mechanical and biological properties (Kumar et al., 2018; Tavangar et al., 2018).

Recent research indicates that the magnetic field generated by nanostructures that have magnetic properties promotes mineralization, cohesion, proliferation, and cell differentiation (Fan et al., 2020). Along with influencing biomineralization behavior during the early stages of gene expression and protein synthesis, the magnetic field affects the structure and crystallization of biomineral products. Also, it alters the spatial structure of proteins in the cytoskeleton. Thus, magnetic nanostructures can adhere to cell surfaces, regulating cell function and increasing bone cell activity, resulting in tissue regeneration. Magnetic nanoparticles primarily comprise cobalt, iron, or nickel that can produce a magnetic field directly and indirectly (Peng et al., 2019). Zinc is a critical nutrient that plays a role in growth, calcium metabolism, ALP activity, and bone metabolism. The absence or inadequacy of these chemical agents retards bone development (Ramezanifard et al., 2016; Laurenti and Cauda, 2017; Peng et al., 2019). Zinc oxide (ZnO) nanostructures have been studied for their potential to enhance cell adhesion, proliferation, and differentiation. Additionally, ZnO characteristics uniquely function in cellular drift, the opening of Ca^2+^ channels in the plasma membrane of osteoblast cells, intracellular calcium transfer, proliferation, and the effect of reactive oxygen species (ROS) on blood vessel development (Laurenti and Cauda, 2017).

In this study, to improve the structure and performance of the scaffold, NiFe_2_O_4_/ZnO-coated PLLA was used as a scaffold to evaluate the potential osteogenic differentiation of hAMSCs, aimed at the regeneration of bone tissue in *in-vivo* conditions.

## 3 Materials and methods

### 3.1 Scaffold synthesis

#### 3.1.1 Fabrication of poly l-lactide scaffold

The electrospinning (Nanoazma, ESI-I, Iran) process was used to fabricate PLLA nanofibers. Briefly, 12% (wt/vol) solution of PLLA (Sigma-Aldrich, MO, United States) in chloroform (Merck, Germany) and dimethylformamide (DMF; Merck, Germany) was drawn into a 5 ml syringe with a 21-gauge needle. Two nozzles were set at an injection rate of 1 ml/h at a distance of 15 cm from the collector. Then, nanofibers were collected on a cylindrical collector with a rotational speed of 600rpm and voltage of 20 kV. Finally, the sheet with a relative thickness of about 200 μm was vacuumed to evaporate the remaining chloroform solution completely.

#### 3.1.2 Scaffolds surface modification

A Plasma quartz reactor (Diener Electronics, Ebhausen, Germany) was applied to induce hydrophilicity in a hydrophobic PLLA scaffold using a low-frequency plasma generator at 90 GHz. The scaffold was put in the reactor chamber, and a vacuum was generated using a dual trap vacuum pump before performing a glow discharge. The PLLA scaffold was subjected to pure oxygen at 0.4 mBar pressure and a flow rate of 10 ml/min for 3 min.

#### 3.1.3 Water contact angle

WCA was determined before and after plasma treatment using a goniometer (GO EDMUND Optic, United States) to evaluate scaffold surface hydrophilicity. PLLA scaffold before and after plasma treatment received one drop of deionized water at room temperature. The images were taken after 10 s, and the contact angle was measured by ImageJ software (NIH United States).

#### 3.1 4. Coating nanofibers with nanocomposite

The NiFe_2_O_4_/ZnO nanocomposite was synthesized using the solid-state method. Briefly, the thiourea was extensively mixed with ZnO and nickel ferrite nanoparticles and then calcined at an 800°C (Yeganeh et al., 2020). After preparation, the nanoparticle powder was dissolved in deionized water at a concentration of (0.1% wt/vol). Then, the solution was placed in an ultrasonic bath for 30 min at 37°C to disperse. The plasma-treated scaffold was immersed overnight in a nanocomposite solution, rinsed twice with deionized water, and dried in a vacuum.

### 3.2 Scaffold characterization

#### 3.2.1 Attenuated total reflectance fourier-transform infrared spectroscopy

The vibrational spectrum of NiFe_2_O_4_/ZnO-coated PLLA and PLLA scaffolds were determined using ATR-FTIR spectroscopy (PerkinElmer-Frontier, United States) with a reading range of 400–4000 cm^−1^ that used a DTGS detector and diamond ATR crystal. PerkinElmer Spectrum version 10.03.06 (PerkinElmer-Frontier, United States) was used to analyze the data.

#### 3.2.2 Tensile test

The SANTAM (STM-20, Iran) device was used to evaluate the mechanical properties of electrospun scaffolds before and after plasma treatment with nanocomposite loading. The scaffolds were carved into a rectangular shape with dimensions of 1 cm × 4 cm, a gauge length of 2 cm, and an 80 μm thickness. Then, it was inserted into the device at room temperature at 5 mm/min crosshead speed. The calculation of tensile was determined by SANTAM machine controller software (STM- 20, Iran).

#### 3.2.3 Scanning electron microscopy

The microstructure of NiFe_2_O_4_/ZnO coated PLLA and the morphology of a PLLA scaffold surface was analyzed. The cell-free scaffolds do not require preparation. Both scaffolds were mounted to an aluminum sample holder with conductive adhesive tape and coated with gold using a KYKY SBC12 sputtering machine at 1 kV and 10 mA for 120 s. Samples were visualized by SEM (AIS2700, SERON technology, South Korea) at 20 kV. The scale of selected SEM images was set to analyze the diameters, then 60 fibers and 10 particles were chosen randomly, and the diameters were measured with ImageJ software (NIH, United States).

### 3.3 Isolation of human adipose tissue-derived mesenchymal stem cell

Adipose tissue was taken from a patient undergoing liposuction surgery in Omid Hospital, Amol, Iran, according to the guidelines of the Medical Ethics Committee, Babol University of Medical Sciences and Health Services (approval code: IR. MUBABOL.HRI. REC 1398.137). Written informed consent was obtained from the next of kin of a 36-year-old man participant for the publication of any potentially identifiable data included in this article. The tissue was transported to the lab under sterile conditions using a solution containing Dulbecco’s Modified Eagle’s Medium (DMEM, Gibco) and penicillin/streptomycin (Pen-strep) (Gibco, United States). Then the tissue was rinsed multiple times with sterile phosphate-buffered saline (PBS, Zistmavad, Iran) containing Pen-strep to eliminate any remaining blood. After discarding the PBS, the adipose tissue was incubated with 0.1% collagenase type I (Gibco, United States) at 37°C for 1 h (hour) in a shaker incubator and then centrifuged at 1200 g for 10 min. The supernatant was discarded, and the pellet was suspended in 10 ml of high glucose DMEM (HG-DMEM, Gibco) supplemented with 1% Pen-strep and 10% fetal bovine serum (FBS, Gibco, United States). Then transferred to a T75 cell culture flask; incubated at 37°C and CO_2_ concentration of 5%. The leftover blood cells were eliminated after 24 h by changing the medium. The culture medium was replaced with fresh medium every 3 days. In this article, basal media refers to HG-DMEM without supplementary material, culture media refers to HG-DMEM with 10% FBS, and osteogenic media (HG-DMEM, which contains 10% FBS, Pen-strep, 10 mM β-glycerophosphate (Sigma-Aldrich, United States), 0.2 mM ascorbic acid (Sigma-Aldrich, United States), and 0.1 µM dexamethasone (Sigma-Aldrich, United States).

### 3.4 Human adipose tissue-derived mesenchymal stem cell characterization

In the second passage, the hAMSCs surface markers, i.e., CD90, CD105, CD45, and CD34, were characterized by flow cytometry. The cells were detached from the T25 culture flask with trypsin-EDTA (Gibco, United States) and centrifuged at 1500 rpm for 5 min. Then the final volume of cell sediment reached 1 ml with PBS. 5 µl of anti-CD90-APC (BioLegend, United States), anti-CD45-FITC (BD Bioscience, United Kingdom), anti-CD105-PE (BioLegend, United States), anti-CD34-PE (IQ Product, United States) were added to 100 µl of cell suspension of each flow cytometry tube and incubated for 30 min at 4°C. Afterward, 500 µl of PBS was added to each tube and centrifuged at 1500 rpm for 5 min. Finally, 250 µl PBS was added to each tube, and cell suspensions were read by BD FACS Calibur (BD Biosciences, United States) and analyzed with Flowjo software version 10.5.3.

### 3.5 Cell culture, adhesion, and differentiation

The effect of PLLA and NiFe_2_O_4_/ZnO coated PLLA scaffolds on cell adhesion, and osteogenic differentiation was examined. On the fourth day after plasma treatment, both Scaffolds were punched to a diameter of 16 mm and subjected to ultraviolet light (UV) for 20 min, then immersed in 70% alcohol for 2 h and rinsed 3 times with sterile PBS to remove any traces of alcohol, then incubated overnight with FBS to enhance cell adhesion. To determine the adherence and proliferation capacity, 2 × 10^4^ hAMSCs were suspended in 100 µl of culture media and seeded on PLLA and NiFe_2_O_4_/ZnO-coated PLLA; 1 h after seeding the cells, the culture media was added to fill each well to the specified volume. After 7 days of culture, the medium was removed and rinsed with PBS. Then, 2.5% Glutaraldehyde (Merck, Germany) in distilled water was added to each well to cover the surface and left over for 1 h. Then scaffolds were dehydrated with 50%–90% methanol solution series left overnight under laminar flow at room temperature to dry and kept in a desiccator.

Additionally, to assess hAMSC differentiation, 2 × 10^4^ cells were seeded in PLLA and NiFe_2_O_4_/ZnO-coated on PLLA. After 24 h, the culture medium was replaced with an osteogenic medium (described above), and every 3 days were changed. On day 21 of differentiation, cells were fixed according to the above procedure; then scaffolds were mounted to an aluminum sample holder with conductive adhesive tape and coated with gold using a KYKY SBC12 sputtering machine at 1 kV and 10 mA for 120 s. Samples were visualized using an SEM (AIS2700, SERON technology, South Korea) at 20 kV.

### 3.6 Bioassays

#### 3.6.1 MTT assay

On days 1, 4, and 7, the metabolic activity of hAMSCs on PLLA, NiFe_2_O_4_/ZnO-coated PLLA, and TCP scaffolds were used to examine. 2 × 10^4^ cells were seeded with a culture medium for this purpose. After discarding the culture media, basal media (described above) containing 50 µl of MTT solution (3-(4,5-Dimethylthiazol-2-yl)-2,5-diphenyl Tetrazolium Bromide) (Sigma-Aldrich, United States) (5 mg/ml MTT in HG-DMEM) was added to each well and incubated at 37°C for 3.5 h. Then, dark-blue intracellular formazan crystals were dissolved in 200 μl dimethyl sulfoxide (DMSO; Sigma-Aldrich, United States) and vortexed for 8 min. The optical density (OD) was determined using a microplate reader (Bio-Tek Instruments, Winooski, VT, United States) at a wavelength of 570 nm.

#### 3.6.2 DAPI staining

DAPI staining was used to determine cell adhesion. For this aim, 2 × 10^4^ hAMSCs were seeded on NiFe_2_O_4/_ZnO-coated PLLA, PLLA, and TCP and cultured in a culture medium, then incubated at 37 °C and 5% CO_2_. After 7 days of incubation, each group was washed twice with PBS and incubated with 2.5% Glutaraldehyde for 1 h. Then glutaraldehyde was removed and washed with PBS. 50 μl DAPI solution (5 μg/ml in diH_2_O; Sigma-Aldrich, Germany) was added to each well of groups and incubated for 5 min. To remove excess and unbounded DAPI stains, they were washed with PBS. The plate was covered and left in the dark, and images were captured using an Immunofluorescence Microscope (Labomed, United States). The scale and threshold of the photos were set and processed. Then, the number of stained cells was determined by analyzing the particle option *via* ImageJ software (NIH, United States).

#### 3.6.3 Alkaline phosphatase activity assay

On days 7, 14, and 21, 2 × 10^4^ hAMSCs were seeded on PLLA, NiFe_2_O_4_/ZnO-coated PLLA, and TCP in the presence of osteogenic media (described above) to determine ALP enzyme activity. The culture media was removed on the appointed days, then rinsed with PBS. After adding 200 μl of radioimmunoprecipitation assay (RIPA) lysis buffer to each well, gently agitated, and cell lysate was centrifuged at 1500 g for 15 min at 4°C. After centrifugation, the supernatant containing total proteins was collected. ALP activity was determined using a procedure provided with an ALP assay kit (Pars Azmoon, Iran). A microplate reader (Bio-Tek Instruments, Winooski, VT, United States) was used to determine the fluorescence intensity at 405 nm. Finally, the enzyme activity (IU/L) was adjusted to the total protein concentration (mg/dl).

#### 3.6.4 Calcium content assay

On days 7, 14, and 21, in the presence of the osteogenic media (described above), the calcium content of hAMSC was determined using the O-cresolphthalein method on PLLA, NiFe_2_O_4/_ZnO-coated PLLA, and TCP. The medium was removed, and each well was rinsed with PBS for calcium extraction. Then, 0.6 N HCL (Merck, Germany) was added to each well and incubated for 1 h by mild shaking at 4°C. Then, the reagent buffer of the calcium content assay kit (Pars Azmoon, Iran) was added and allowed for incubation. A microplate reader (Bio-Tek Instruments, Winooski, VT, United States) read the OD of samples at 570 nm. The standard curve was generated using repeated dilutions of 0.1 M calcium chloride solution.

#### 3.6.5 Alizarin-red staining

To assess mineral sediments formed as a result of differentiation, on days 7, 14, and 21, 2 × 10^4^ cells were seeded on PLLA, NiFe_2_O_4_/ZnO-coated PLLA, and TCP scaffolds in the presence of osteogenic media (explained above). After removing the culture media and washing with PBS, the cells were fixed with the same procedure described above. Each well received 200 µl Alizarin red (Sigma-Aldrich) solution (2 mM in deionized water (diH_2_O) and incubated at room temperature for 15min by mild agitation, then rinsed with PBS several times until the color was removed entirely and only a purple halo remained. Ultimately, the photos were captured using a stereomicroscope (Olympus, Japan).

### 3.7 Quantitative-Real-time PCR

The real-time PCR was used to determine the mRNA expression levels of *Osteonectin, Osteocalcin, ALP, Collagen type I,* and Runt-related transcription factor 2(*Runx2*) genes on days 7, 14, and 21 of the culture on scaffolds. The primer sequences used for qRT-PCR are shown in table1. First, RNA was isolated from hAMSCs cultured on TCP, PLLA, and NiFe_2_O_4_/ZnO-coated PLLA scaffolds in osteogenic media using the RNA extraction kit (MaxSpin, Maxcell, Iran). To synthesize cDNA, 5 µg of RNA was mixed with 2 µl of BON-RTmix primer (1 mM) (Bonbiotech, IRAN), 11 µl of DEPS water, 1 µl of BON-RT enzyme (5U/ml; Bonbiotech, IRAN), 3 µl of dNTP mix (Bonbiotech, Iran), and 6 µl RT buffer (Bonbiotech, Iran). The samples were incubated in a thermocycler (Bio-Rad) for 10 min at 25°C, 15 min at 37°C, 45 min at 42°C, and 10 min at 72°C, respectively. Then, 6.5 µl of master mix SYBR green 2x (BON-QPCR, Bonbiotech, Iran), 1 µl of specific F and R primers, 1 µl of cDNA, and 0.25 µl ROX Reference Dye were combined and incubated for 2 min at 95°C, followed by 5 s at 95°C and 30 s at 60°C for 40 cycles on an ABI Applied Biosystems™ thermal cycler (Thermo Fisher, United States). The relative expression of genes was determined by the 2^−ΔΔCT^ method. The *B2-microglobulin* gene was used as the internal control. Also, Rest software 2009 was used for analyzing the data.

**TABLE 1:** Primers used in Real-time PCR.

Gene name	Primer sequences	Product size (bp)	
*β-2-Micro globulin* (*β2M*)	Forward	5′-TGG​AAA​GAA​GAT​ACC​AAA​TAT​CGA-3′	201
	Reverse	5′-GAT​GAT​TCA​GAG​CTC​CAT​AGA​GCT-3′	
*Osteonectin*	Forward	5′-AGG​TAT​CTG​TGG​GAG​CTA​ATC-3′	224
	Reverse	5′-ATT​GCT​GCA​CAC​CTT​CTC-3′	
*Osteocalcin*	Forward	5′-GCA​AAG​GTG​CAG​CCT​TTG​TG-3′	86
	Reverse	5′-GGC​TCC​CAG​CCA​TTG​ATA​CAG-3′	
*Alkaline phosphatase* (*ALP*)	Forward	5′-GCA​CCT​GCC​TTA​CTA​ACT​C-3′	162
	Reverse	5′-AGA​CAC​CCA​TCC​CAT​CTC-3′	
*Collagen I*	Forward	5′-TGG​AGC​AAG​AGG​CGA​GAG-3′	121
	Reverse	5′-CAC​CAG​CAT​CAC​CCT​TAG​C-3′	
*Runx2*	Forward Reverse	5′-GCC​TTC​AAG​GTG​GTA​GCC​C-3′ 5′-CGT​TAC​CCG​CCA​TGA​CAG​TA-3′	86

### 3.8 Statistical analysis

All experiments were performed in triplicate, and mechanical experiments were performed with *n* = 6. Obtained data were shown as mean ± SD. The MTT assay, ALP enzyme, calcium content, and gene expression were analyzed *via* two-way analysis of variance (ANOVA), and DAPI staining was analyzed *via* one-way ANOVA. Also, Tukey’s multiple comparisons tests were used for means that are significantly different from each other in all analyses by GraphPad Prism software version 8.3.1 and Microsoft Excel software version 16.11.1. Also, the p-value was used to show statistical significance (*p* < 0.05 was considered significant).

## 4 Result

### 4.1 Morphology and microstructure of electrospun nanofibers

**FIGURE 1:**
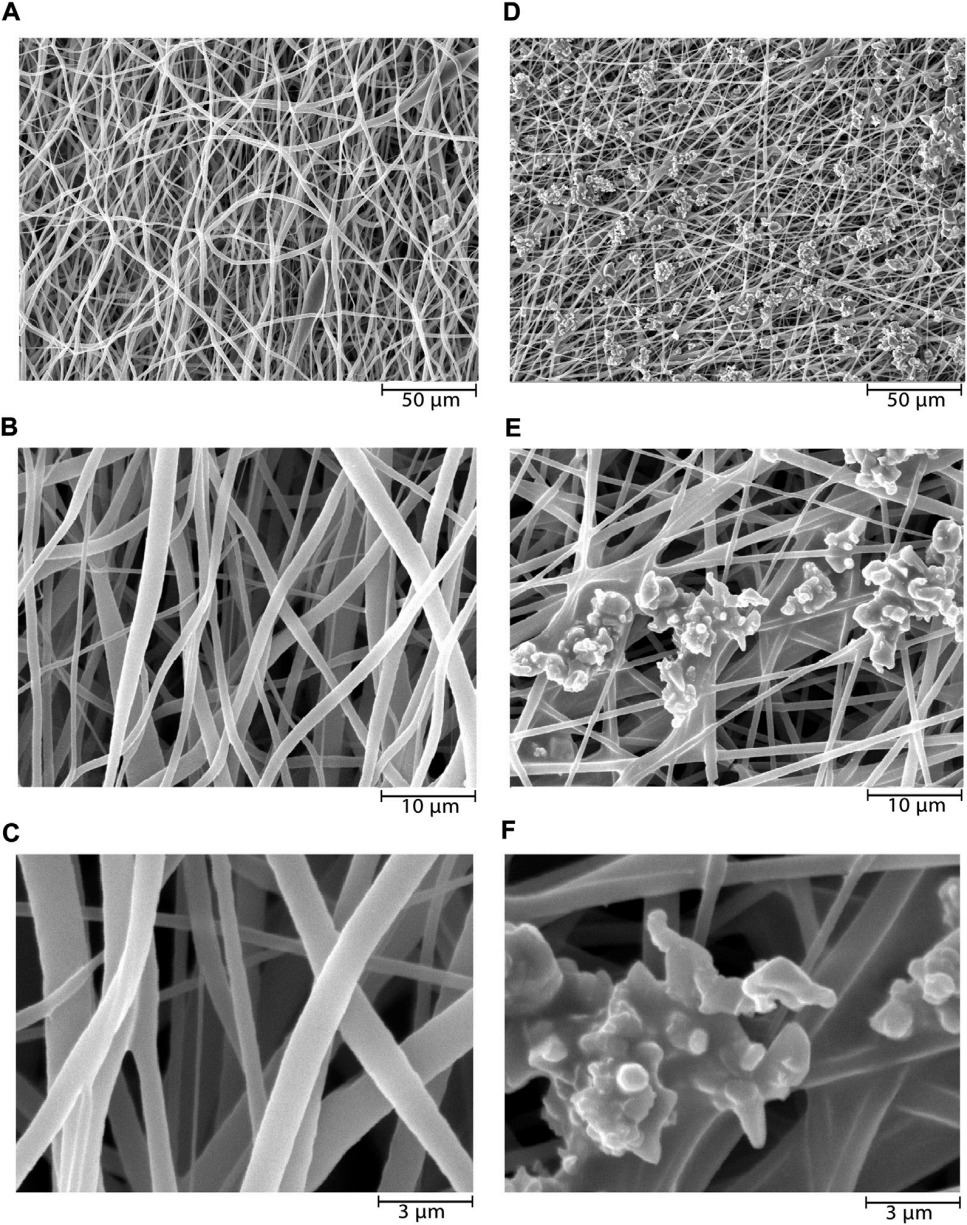
SEM microstructure of electrospun PLLA nanofibers before and after nanocomposites deposition. PLLA nanofibers at ×1000 **(A)**, ×5000 **(B)**, and ×15,000 **(C)** magnifications, and NiFe_2_O_4_/ZnO-coated PLLA nanofibers at ×1000 **(D)**, ×5000 **(E)**, and ×15,000 **(F)** magnifications.

In this study, SEM micrographs of nanofibers morphology depict entirely random and nearly homogenous fibers which have lacked a bead and porous cavities (Figure 1A–C). After coating the NiFe_2_O_4_/ZnO on the PLLA scaffold with a mean size of 83 nm ± 9.01, a nearly homogeneous distribution of nanocomposite was found on the surfaces. However, it may have an aggregation number. The nanocomposite had a uniform aggregated spherical morphology with some voids (Figure 1D–F). The mean diameters of nanofibers were about 678 nm ± 7.43 when measured using ImageJ software (NIH United States). The diameter of nanofibers did not change after coating the nanocomposite (Figure 1D–E).

Hydrophilicity was determined by measuring the water contact angle of PLLA nanofibers before and after plasma treatment. This angle was 117° before plasma treatment and decreased to 0° following plasma treatment. Before plasma treatment, the tensile strength of the PLLA nanofiber scaffold was 0.36 ± 0.18 MPa, and elongation at peak break was 67.26%. Still, after plasma treatment and nanocomposite presence, the tensile strength increased to 1.21 ± 0.41 Mpa, and elongation at break peak was 53.54%. As a result, the PLLA scaffold’s mechanical characteristics were enhanced after plasma treatment and coating of the nanocomposite.

**FIGURE 2:**
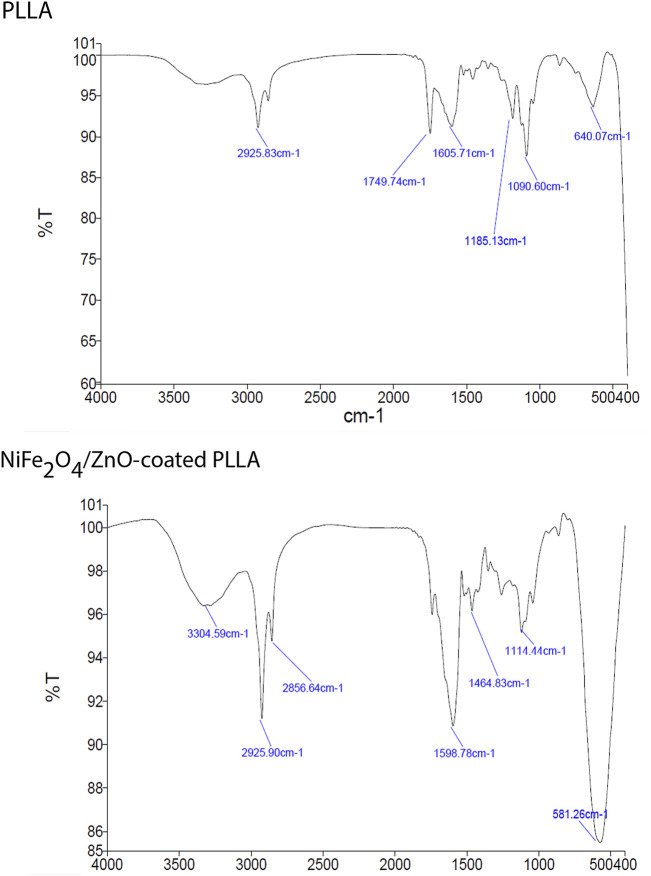
ATR-FTIR spectra of PLLA and NiFe_2_O_4_/ZnO-coated PLLA scaffolds.

Due to the vibrations of PLLA nanofibers described previously (Ramezanifard et al., 2016), FTIR measurements revealed peaks at 1749.74 cm^−1^ corresponding to C=O tensile of the carbonyl group,1090.60 cm^−1^ corresponding to C—O antisymmetric stretching, and 1185.13 cm^−1^ corresponding to (C-O-C stretching). The O-H vibration has a broad peak at 3304.59 cm^−1^. The vibration at 581.26 cm^−1^ is a stretching vibration of oxygen metal (Fe^3+^-O^2-^) or a combination of ZnO and ferrite stretching. Nanocomposite’ OH bending vibrations cause the peak at 1114 cm-1. The increase in the strength of vibrations in this group confirms the presence of nanocomposite on PLLA (Figure 2).

### 4.2 Characterization, adhesion, and differentiation of human adipose tissue-derived mesenchymal stem cell on scaffolds

**FIGURE 3:**
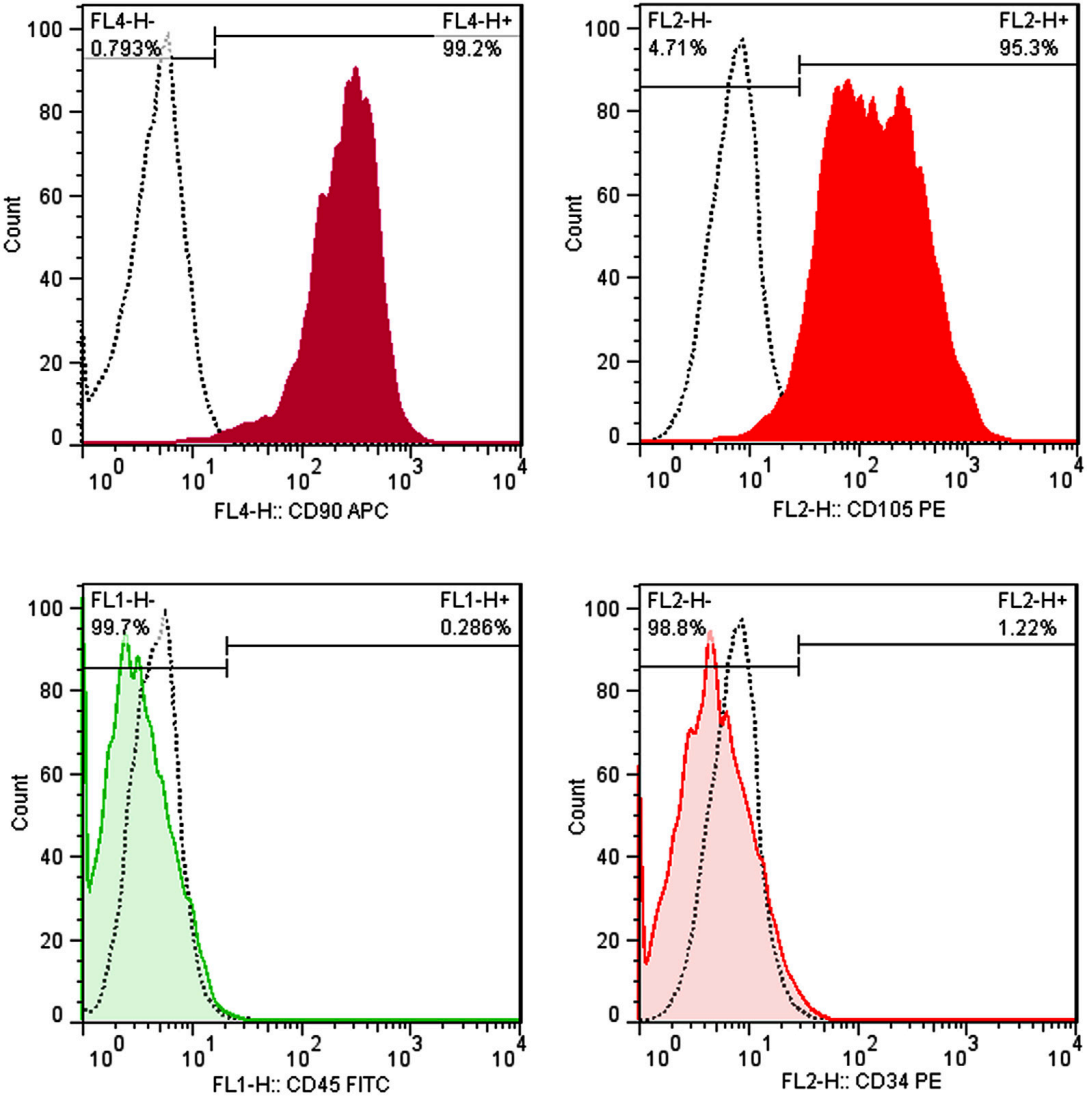
Immunophenotyping of hAMSCs using flow cytometry. hAMSCs were positive for CD105 and CD90 and negative for CD34 and CD45. (dotted curve: unstained samples, color curve: stained samples).

As shown in Figure 3, positive markers (CD105, CD90) have an expression level of more than 95% of cells when surface antigens were analyzed by flow cytometry. Additionally, the hematopoietic markers CD34 and CD45 were expressed at 1.22% and 0.29%, respectively, confirming that the separated cells’ have a mesenchymal origin.

**FIGURE 4:**
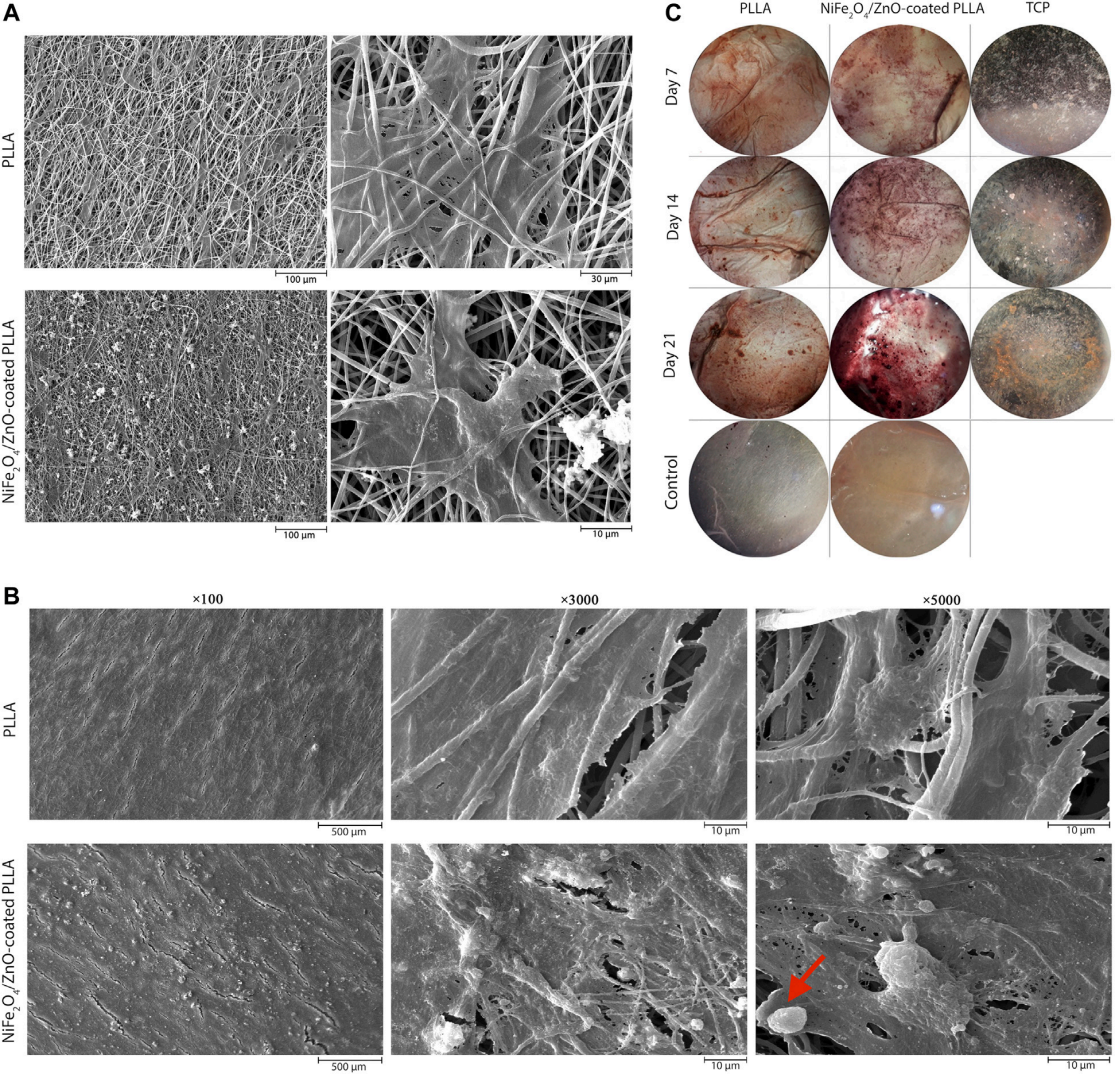
Adhesion, proliferation, differentiation and staining of hAMSCs on PLLA. SEM microstructures of adhesion on day 7**(A)** and differentiation on day 21 **(B)** of hAMSCs on PLLA and NiFe_2_O_4_/ZnO-coated PLLA. **(C)** Alizarin-red staining of hAMSCs on different substrates (cell-free scaffolds considered a control group).

On day 7, adhesion and proliferation of hAMSCs on PLLA and NiFe_2_O_4_/ZnO-coated PLLA scaffolds demonstrates the distribution of cells on the scaffold surface, indicating proper contact and integration of cells with PLLA and NiFe_2_O_4_/ZnO-coated PLLA scaffolds have occurred (Figure 4A). On both types of scaffold surface, cell adhesion and proliferation are seen, suggesting the low toxicity of PLLA and NiFe_2_O_4_/ZnO-coated PLLA scaffolds (Figure 4A).

After 21 days of differentiation, the mineral deposits on NiFe_2_O_4_/ZnO-coated PLLA were much more significant than PLLA. The deposition of calcium and hydroxyapatite granules on the surface of nanofibers was discovered to have a significant association with mineral mass. Mineral deposits shown in the red arrow (Figure 4B) seem to have a porous and rough shape at high magnification; this appearance may be due to the accumulation of sediments on top of each other, which shows spherical aggregates of minerals. Sedimentation occurred at a meager rate in PLLA (Figure 4B).

### 4.3 Bioassays

#### 4.3.1 Alizarin-red staining (biomineralization)

ARS was utilized to assess the osteogenic differentiation of hAMSC on different surfaces qualitatively. The amount of alizarin-red dots associated with mineral deposits and calcium increased on days 14 and 21. On the 21st day of differentiation, the largest values of these sediments were observed on the NiFe_2_O_4_/ZnO-coated PLLA scaffold. Additionally, there was no color on cell-free scaffolds (control), indicating that PLLA and NiFe_2_O_4_/ZnO are achromatic in the absence of cells (Figure 4C).

#### 4.3.2 Calcium content

**FIGURE 5:**
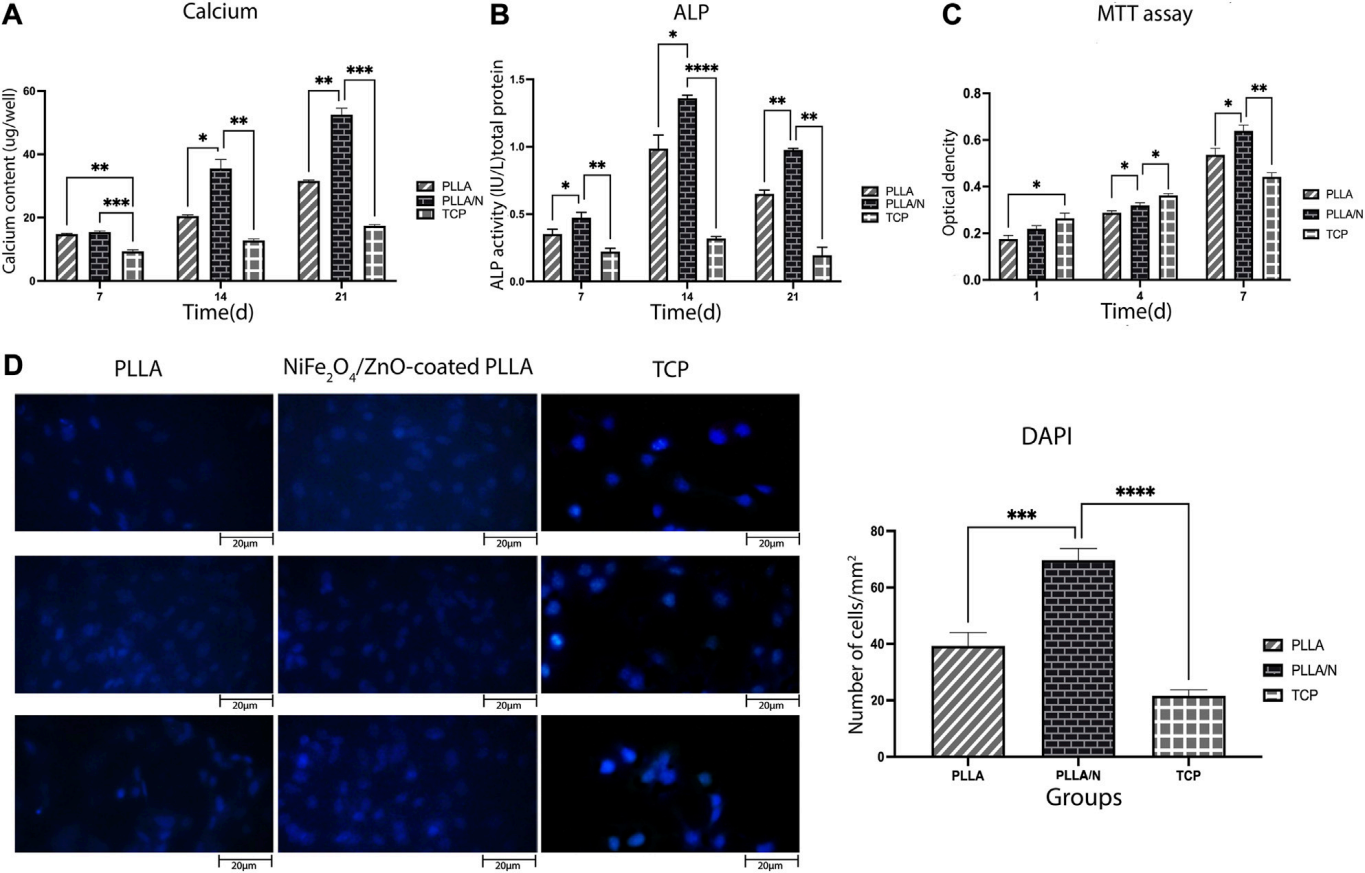
Bioassays in the osteogenic differentiation process. Mineralized calcium deposition **(A)** and Alkaline phosphatase (*ALP*) activity of osteogenic differentiation of hAMSCs on different scaffolds **(B)** Cell viability of hAMSCs on different scaffolds **(C)** and DAPI staining of hAMSCs cultured on different substrates**(D)** (mean ± SD; *p*-value < 0.05). (*p < 0.01, **p < 0.001, ***p < 0.0001, ****p < 0.0001).

On days 7, 14, and 21, the calcification rate was determined in an osteogenic environment. On days 14 (PLLA = 20.50 ± 0.36, PLLA/*n* = 35.53 ± 2.80, TCP = 12.80 ± 0.45) and 21, the calcium concentration of the NiFe_2_O_4_/ZnO-coated PLLA scaffold was higher than that of the PLLA and TCP scaffolds (*p* < 0.05). However, on day 7, no statistically significant variation in calcium concentration was observed between the NiFe_2_O_4_/ZnO-coated PLLA and the PLLA scaffold (PLLA = 14.83 ± 0.20, PLLA/*n* = 15.46 ± 0.32, TCP = 9.33 ± 0.50) (*p* > 0.05). On day 21 of differentiation, a higher calcium level was reported in the NiFe_2_O_4_/ZnO-coated PLLA scaffold than PLLA scaffold and TCP (PLLA = 31.60 ± 0.26, PLLA/*n* = 52.53 ± 2.04, TCP = 17.43 ± 0.35) (Figure 5A).

#### 4.3.3 Alkaline phosphatase

ALP activity indicates early osteoblastic differentiation and commitment of stem cells to the osteoblastic phenotype. Results showed that ALP activity has increased from day 7(PLLA = 0.3 ± 0.035, PLLA/*n* = 0.4 ± 0.3, TCP = 0.22 ± 0.02) to day 14 (PLLA = 0.98 ± 0.1, PLLA/*n* = 1.36 ± 0.02, TCP = 0.31 ± 0.01) in cells cultured on both scaffolds. Each day, the mean absorption ALP activity in NiFe_2_O_4_/ZnO-coated PLLA scaffold was higher than PLLA and TCP (*p* < 0.05). On day 21, the ALP enzyme activity decreased in all groups (PLLA = 0.64 ± 0.03, PLLA/*n* = 0.97 ± 0.01, TCP = 0.19 ± 0.06) (Figure 5B).

#### 4.3.4 Human adipose tissue-derived mesenchymal stem cell viability on different substrates

The viability and adherence of hAMSCs on different scaffolds were assessed with MTT assay (on days 1, 4, and 7 after seeding) and DAPI staining (on day 7). On days 1 )PLLA = 0.17 ± 0.016, PLLA/*n* = 0.21 ± 0.014 , TCP = 0.26 ± 0.022 ) and 4 (PLLA = 0.28 ± 0.009 ,PLLA/*n* = 0.32 ± 0.01,TCP = 0.36 ± 0.008) the cell viability (proliferation) was higher on TCP compared to PLLA and NiFe_2_O_4_/ZnO-coated PLLA scaffolds. Still, on day 7(PLLA = 0.53 ± 0.028, PLLA/*n* = 0.63 ± 0.02, TCP = 0.44 ± 0.018), most of the cell’s metabolic activity occurred on the NiFe_2_O_4_/ZnO-coated PLLA scaffold (*p* < 0.05) (Figure 5C).

On day 7, hAMSCs cultivated on NiFe_2_O_4_/ZnO-coated PLLA scaffold had a higher population density per mm^2^ surface area than TCP and PLLA scaffold (*p* < 0.05). It provides a bioactive and biocompatible environment where cells can adhere and proliferate (PLLA = 39.33 ± 4.7, PLLA/*n* = 69.66 ± 4.16 TCP = 21.66 ± 2.08) (Figure 5D).

## 5 Osteogenic gene expression

To evaluate the osteogenic differentiation in cells cultured on scaffolds and TCP, the expression of critical osteogenic genes (*Osteonectin, Osteocalcin, ALP, Runx2, and Collagen type I*) were measured. On all days (Ghobeira et al., 2017; Porgham Daryasari et al., 2019; Yeganeh et al., 2020; Yeganeh et al., 2020), a higher expression of the *ALP gene* was observed in the differentiation on NiFe_2_O_4_/ZnO-coated PLLA scaffold compared to TCP and PLLA (*p* < 0.05) (day7; PLLA = 0.94 ± 0.0, PLLA/*n* = 1.11 ± 0.03, and TCP = 0.72 ± 0.14).

The transcription of the *ALP* gene during differentiation was more on day 14 (PLLA = 1.24 ± 0.16, PLLA/*n* = 2.87 ± 0.09, and TCP = 1 ± 0.03) than on day 21(PLLA = 0.93 ± 0.03, PLLA/*n* = 1.32 ± 0.17, and TCP = 0.70 ± 0.19). On day 7(PLLA = 0.95 ± 0.04, PLLA/*n* = 1.21 ± 0.09, and TCP = 0.73 ± 0.07) Day 14 (PLLA = 1.57 ± 0.08, PLLA/*n* = 1.91 ± 0.07, TCP = 1.11 ± 0.05) to 21, the expressions of *Osteonectin* on NiFe_2_O_4_/ZnO-coated PLLA scaffold was increased compared to the PLLA and TCP (*p* < 0.05). On day 21 of differentiation, osteonectin gene expression decreased in PLLA and TCP but increased on NiFe2O4/ZnO-coated PLLA scaffold (PLLA = 1.50 ± 0.19, PLLA/*n* = 2.67 ± 0.09, and TCP = 1.02 ± 0.23). Regarding the expression of the *Osteocalcin* in this study, no significant statistical difference was observed in all three groups on day 7 (PLLA = 1.1 ± 0.18, PLLA/*n* = 1.31 ± 0.10, and TCP = 1 ± 0.01) (*p* > 0.05). In contrast, on days 14 (PLLA = 1.20 ± 0.04, PLLA/*n* = 1.9 ± 1.06, and TCP = 1.0 ± 0.15) and 21(PLLA = 1.61 ± 0.2, PLLA/N = 2.9 ± 0.03, and TCP = 1.04 ± 0.31), the expression of *Osteocalcin* was much increased in cultured cells on NiFe_2_O_4_/ZnO-coated PLLA scaffold compared to PLLA and TCP (*p* < 0.05). On days 14 (PLLA = 1.17 ± 0.02, PLLA/N = 1.34 ± 0.04, and TCP = 1.09 ± 0.0) and 21, the expression of the *Runx2* gene in cultured cells on the NiFe_2_O_4_/ZnO-coated PLLA scaffold was more than PLLA and TCP (*p* < 0.05). However, on day 7(PLLA = 1.0 ± 0.01, PLLA/*n* = 1.08 ± 0.0, TCP = 1.07 ± 0.01), there was no significant statistical difference in *Runx2* gene expression between the three groups. On day 21(PLLA = 1.2 ± 0.15, PLLA/N = 2.44 ± 0.14, and TCP = 1.01 ± 0.21) of differentiation, the expression of this gene increased in cultured cells on the NiFe_2_O_4_/ZnO-coated PLLA scaffold. The level of *Runx2* gene expression has remained almost constant for PLLA and TCP; a slight downward trend was observed.

On days 7(PLLA = 1.13 ± 0.0, PLLA/N = 1.25 ± 0.01, and TCP = 1 ± 0.09) and 14 (PLLA = 1.11 ± 0.02, PLLA/*n* = 1.27 ± 0.12, and TCP = 1 ± 0.04) of differentiation, there was no significant difference in the *Collagen type I* gene expression level between all three groups (*p* > 0.05). Still, on day 21, the expression of the *Collagen type I* in cultured cells on NiFe_2_O_4_/ZnO-coated PLLA scaffold was increased compared to the PLLA and TCP (PLLA = 1.31 ± 0.1. PLLA/*n* = 1.98 ± 0.11, and TCP = 1.18 ± 0.13) (*p* < 0.05).

## 6 Discussion

Bone tissue engineering aims to create materials that enter irreparable bone tissue lesions and regenerate through resident cells. Biomaterials can mimic the ECM’s function, resulting in cellular and vascular infiltration and structure of the bone matrix in injured tissue (Laurenti and Cauda, 2017; Koons et al., 2020; Troy et al., 2021). Numerous studies have been conducted to determine the best ability of nanofiber scaffolds incorporating nanoparticles, such as bioceramic, metal, etc., to repair bone defects. Even without bone growth agents, nanostructured materials operate as an effective signal in the mechanism of osteoblastic differentiation (Dang et al., 2018; Petersen et al., 2018; Tavangar et al., 2018). On the other hand, ideas such as bone protein uptake, topography, and surface calcification are proposed for assessing the ossification function of chemicals that can cause it (Tavangar et al., 2018; Montoya et al., 2021).

The ability of hAMSCs to differentiate into osteogenic cells was evaluated in this experiment using NiFe_2_O_4_/ZnO-coated PLLA as a scaffold to target bone tissue engineering. According to this, the mechanical properties of the PLLA scaffold improved following plasma treatment and nanocomposite coating. This method revealed evidence of additional tensile strength. Plasma therapy leads to carboxyl and hydroxyl groups forming electrostatic bonds with soluble ions such as calcium and growth hormones (Karimi et al., 2019). We hypothesize that this effect is due to the stiffness of NiFe_2_O_4_/ZnO-coated PLLA scaffolds. The stiffness of the scaffold promoted osteogenic proteins (Osteonectin and Osteopontin) and vascularization, resulting in a significant correlation between vascular growth, bone formation, and bone matrix deposition (Karimi et al., 2019; Montoya et al., 2021).

Wettability is essential for cell adhesion, and expansion and sterilization methods may affect it. About 60%–80% ethanol solution is a common disinfection technique that does not affect the chemical and morphological properties of the scaffold, but its high concentrations may cause fiber shrinkage (Ghobeira et al., 2017; Łopianiak and Butruk-Raszeja, 2020). Effective sterilization is ensured by combining ethanol and UV radiation, which is an effective method because the physical and chemical changes caused by H2O2 Plasma (HP) or Ethylene Oxide (ETO) occur far less and maintain the biocompatibility of sterilized nanofibers. In the studies done after UV sterilization, WCA remains constant, which indicates that 3 h of UV exposure does not cause surface chemical changes. Still, long-term UV radiation for 5–24 h can cause drastic differences in topography and chemical composition (Park et al., 2011; Ghobeira et al., 2017).

The nanocomposites applied to the scaffold surface, as shown in (Figure 1D–F), did not obstruct the porosity space of the scaffold. Also, nanocomposites coated on the scaffold surface have no inhibitory effect on adhesion cell proliferation and survival but rather enhance cell proliferation (Figure 4A). Connected pore networks can facilitate the movement of nutrients, oxygen, waste products, and angiogenesis. Roughness on the surface and other topological properties can enhance cell adhesion and fate (Gaharwar et al., 2020).

On day 7, DAPI staining revealed that the number of cells in the NiFe_2_O_4_/ZnO-coated PLLA scaffold was much higher (Figure 5D)We hypothesize that this effect can be attributed to the presence of magnetic nanoparticles to modulate and proliferate hAMSCs. Nanocomposites with magnetic properties, i.e., nickel and ferrite, affect cell proliferation and survival, achieved with surface modifiers. The broad porous nanofibers contain anchors that promote cell attachment and proliferation while boosting cell cohesion and diffusion (Seyedjafari et al., 2010; Vieira et al., 2017; Fan et al., 2020).

Cell growth and proliferation were increased on both scaffolds throughout the 21-day monitoring of hAMSCs differentiation into the osteogenic lineage (Figure 4B) but were higher on the nanocomposites-coated scaffold, which also had more cell layers on the surface. We hypothesized that hydroxyapatite granules on the surface of the NiFe2O4/ZnO-coated PLLA promote differentiation into the osteogenic lineage. Carbonate hydroxyapatite is a bone mineral necessary for forming phosphate and calcium. It is found between collagen fibers. These compounds are responsible for bone hierarchical and mechanical structural properties (Koons et al., 2020). We observed a slight cellular infiltration (Figure 4B), which is unclear in the image due to surface coating with cells. The major limitation of electrospun scaffolds is that they have solid layers with only a single surface porous network, which is limited to the sheet-like formation (Wu and Hong, 2016). Although electrospun scaffolds have high porosity, the pore diameter of these scaffolds is much smaller than the diameter of cells in the micrometer range. This unavoidable feature limits cell penetration through the scaffolds. But the depth of cell penetration into the Electrospun scaffolds varies from approach to approach (Blakeney et al., 2011; Khorshidi et al., 2016). Microporous scaffolds have been shown to promote hAMSC adhesion and osteogenic differentiation. Through intracellular and intercellular signaling, the material’s topographic and biochemical properties can alter the microenvironment of the desired location. These microenvironmental modifications affect cell differentiation by regulating enzymes, cells, and ions containing radical species (Gaharwar et al., 2020).

ALP activity was assessed based on our findings from the process of osteoblastic differentiation of hAMSC on PLLA, NiFe_2_O_4_/ZnO-coated PLLA, and TCP. The most activity of the ALP enzyme (Figure 5) was seen in nanofibers of NiFe_2_O_4_/ZnO-coated PLLA throughout this procedure. We assume this is due to Zinc in this nanocomposite. Zinc acts as a cofactor in ALP activity and promotes the development of osteoblastic activity (Laurenti and Cauda, 2017). ALP activity in osteoblasts is the primary marker of osteogenesis and hard tissue (Jaiswal et al., 2013).

As a significant factor, calcium functions in creating osteogenesis (Khader and Arinzeh, 2020). On the NiFe_2_O_4_/ZnO-coated PLLA scaffold (Figure 5), calcium levels rise during the differentiation phase from day 7 to day 21 due to the support of ALP activity, which initiates the mineralization process. By releasing signaling ions, biodegradable biomaterials can alter the environment. Calcium can excite calcium sensory receptors, essential for cell proliferation, differentiation, and chemotaxis. In addition, releasing ions from the phosphate, calcium, and biomaterial bases can stimulate endogenous cells, causing them to develop into the osteogenic lineage (Gaharwar et al., 2020).

On day 21, the highest amount of calcium mineralization occurred in the NiFe_2_O_4_/ZnO-coated PLLA, as shown in Figure 4C of Alizarin red staining, which serves as a seal of approval for the process of osteoblast cell mineralization and maturation during the differentiation process into the osteogenic lineage. Studies show that the magnetic mechanism of action is mediated through the calcium ion transport channel in the cell membrane. Additionally, it could affect the structure and crystallization of biomineral products, alter the spatial organization of proteins in the cytoskeleton, and affects the biomineralization behavior during the early stages of gene expression and protein synthesis (Fan et al., 2020). Cell receptors, proteins, and peptides interact to enable the cell to store minerals and ECM proteins (Koons et al., 2020).

**FIGURE 6:**
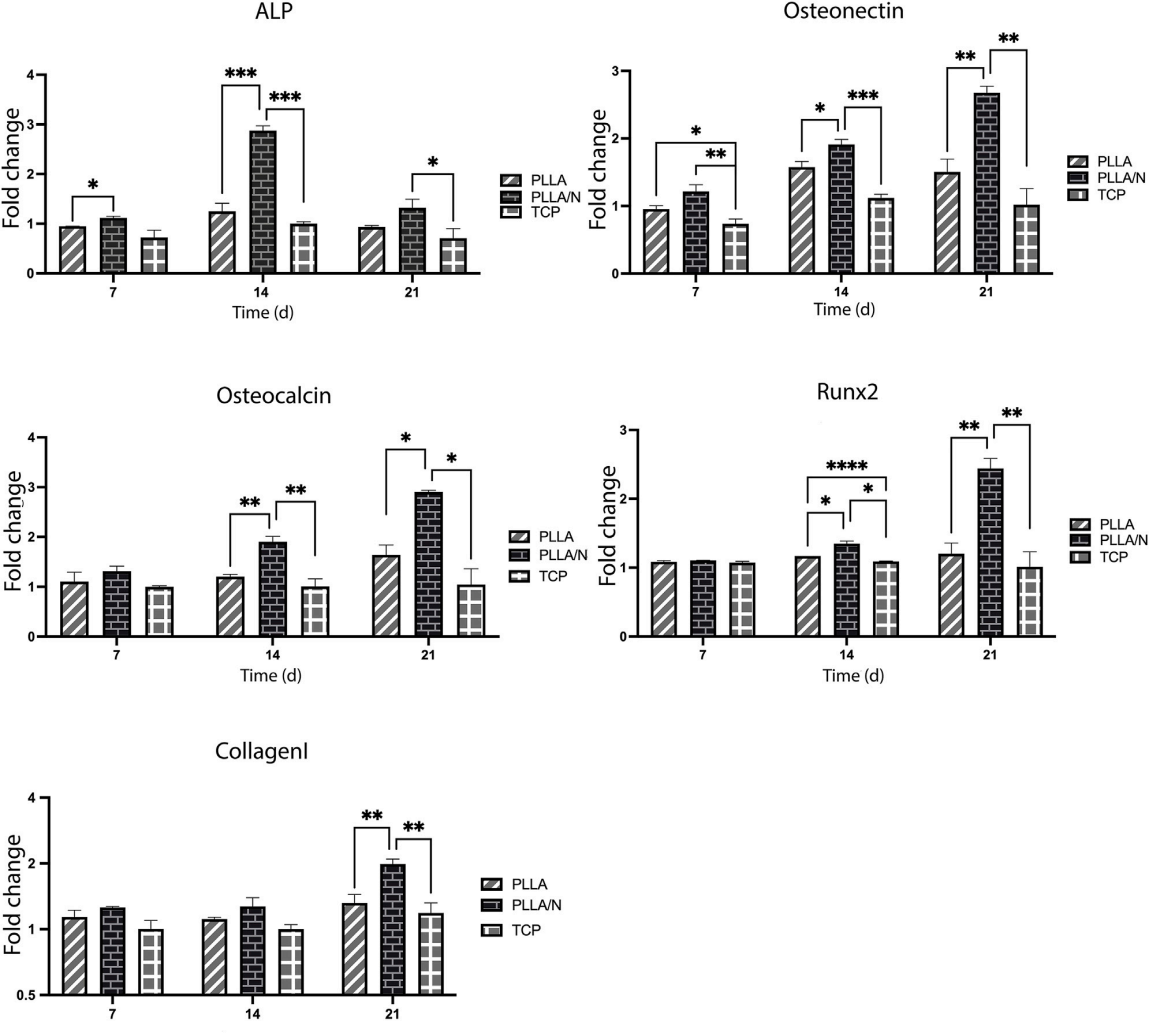
Fold changes of mRNA expression. Alkaline phosphatase (*ALP*), *Osteonectin, Osteocalcin, Runx2*, and *Collagen type I* in osteogenic differentiation of hAMSCs on PLLA, NiFe_2_O_4_/ZnO-coated PLLA. (mean ± SD; *p*-value< 0.05). (*p < 0.01, **p < 0.001, ***p < 0.0001, ****p < 0.0001).

During the 21-day observation of osteogenic lineage development, bone-related mRNAs (*Osteonectin, Osteocalcin, ALP, Runx2, and Collagen type I*) were detected during the molecular behavior of hAMSC. The nanocomposites on the scaffold surface boosted and maintained Runx2 expression until day 21 (Figure 6). The *Runx2* gene regulates the expression of phenotypic markers such as osteocalcin and ALP during the differentiation of hAMSCs into pre-osteoblasts (Abe et al., 2019). The highest level of ALP mRNA expression on day 14 indicated the nanocomposites’ osteoblastic activity; we hypothesize this can be attributed to the action of Zinc, an essential element. ZnO significantly affected the amount of ALP and the magnetic field produced by nickel ferrite, which is effective at interacting with cells and their ossifying role (Ramezanifard et al., 2016; Abe et al., 2019).

The *Runx2* gene expression on the 21st day of differentiation was increased on the NiFe_2_O_4_/ZnO-coated PLLA scaffold. The high expression of the *Runx2* gene was shown to increase the Bone Morphogenetic Protein-9 (Bmp9) gene expression (Karimi et al., 2019). Osteonectin is a calcium-binding glycoprotein that plays a function in the crystallization of osteoblasts during their early stages of development (Nafary et al., 2017). The highest level of Osteonectin gene expression was seen on day 21 on the NiFe_2_O_4_/ZnO-coated PLLA scaffold, which corresponds to the mineralization stage (2 weeks of differentiation) in the previous studies (Seyedjafari et al., 2010; Nafary et al., 2017). The *Osteocalcin* gene is known as a bone-growth protein (BGP) that osteoblasts generate and release during their maturation (3 weeks of differentiation) (Nafary et al., 2017; Nguyen et al., 2019). The highest level of *Osteocalcin* mRNA expression on day 21 indicates the osteoblastic activity of the NiFe_2_O_4_/ZnO-coated PLLA scaffold. We hypothesize that it can be attributed to the magnetic properties of the nanocomposite. *Collagen type I* is the most abundant protein in the bone matrix, as it aids in mineralization during bone formation (Peng et al., 2019). The expression of *Collagen type I* was increased on day 21 in this study, while there was no statistically significant difference between the groups on days 7 and 14 of osteoconductive culture. As a result, they provide surfaces capable of transmitting biological signals by mimicking the structure of the ECM (Chen et al., 2017; Dang et al., 2018; Komori, 2019). Numerous Studies indicated that moving electrons provide magnetic properties to stem cells, aiding their cohesion, survival, migration, proliferation, and differentiation. Their porosity space can result in the formation of arteries, angiogenesis, and a bone-like environment, enabling bone tissue regeneration (Chen et al., 2017; Komori, 2019; Dixon and Gomillion, 2022). Our results indicate that NiFe_2_O_4_/ZnO-coated PLLA and PLLA nanofibers prolong the osteoblastic obligation process in hAMSC.Combining PLLA nanofibers with nanocomposite (NiFe_2_O_4_/ZnO) promotes cell interaction and ossification in an osteogenic environment. The NiFe_2_O_4_/ZnO-coated PLLA scaffold may be used in animal models of bone injury to confirm this work’s findings, consistent with the link between osteogenic function *in vitro* and *in vivo*.

## 7 Conclusion

The differentiation of hAMSC into the osteogenic lineage demonstrated that interaction of cells with NiFe_2_O_4_/ZnO coated PLLA scaffold could provide a support role, like bone ECM architecture, culminating in osteoblast adhesion, proliferation, differentiation, and maturation. We assume that NiFe_2_O_4_/ZnO-coated PLLA could be the proper platform for directing connections and cellular activity toward the osteogenic lineage by hypothesizing having piezoelectric, magnetic, and proper mechanical properties. However, Future studies should be conducted to deepen the study of these properties and the potential of NiFe_2_O_4_/ZnO-coated PLLA scaffold designed as an *in vivo* osteoinduction material.

## Data Availability

The raw data supporting the conclusions of this article will be made available by the authors, without undue reservation.

## Abbreviations

ATR-FTIR, Attenuated Total Reflectance Fourier-Transform Infrared; ALP, Alkaline phosphatase;ARS, Alizarin-red staining; BGP, Bone growth protein; BMP9, Bone morphogenic protein-9; BTE, Bone tissue engineering; DAPI, 4′,6-Diamidino-2-phenylindole dihydrochloride; diH_2_O, deionized water; DMF, Dimethylformamide; DMEM, Dulbecco’s Modified Eagle’s Medium; DMSO, Dimethyl sulfoxide; ECM, Extracellular matrix; FBS, Fetal bovine serum; H, Hour; hAMSCs, human Adipose tissue-derived Mesenchymal Stem Cells; HG-DMEM, High glucose- Dulbecco’s Modified Eagle’s Medium; HP, H2O2 Plasma; EtO, Ethylene Oxide; MTT(3-(4,5-Dimethylthiazol-2-yl)-2,5-diphenyl Tetrazolium Bromide); PLLA, Poly L-Lactide; PBS, Phosphate-buffered saline; Pen-strep, penicillin and streptomycin; RIPA, Radioimmunoprecipitation assay; ROS, Reactive oxygen species; Runx2, Runt-related transcription factor 2; SEM, Scanning Electron Microscopy; OD, Optical density; TCP, Tissue culture plastic; UV, Ultraviolet light; WCA, Water contact angle; ZnO, Zinc oxide.
